# Synthesis of an All-sp^2^‑Hybridized Spheriphane C_48_H_30_

**DOI:** 10.1021/acs.orglett.6c02209

**Published:** 2026-06-08

**Authors:** Jing-Yuan Wang, Chang-Rui Chen, Che-Wei Chang, Cheng-chau Chiu, Kwan Yin Cheung

**Affiliations:** † Department of Chemistry, 34874 National Sun Yat-sen University, Kaohsiung 80424, Taiwan (R.O.C.); ‡ Green Hydrogen Research Center, National Sun Yat-sen University, Kaohsiung 80424, Taiwan (R.O.C.); § Center for Theoretical and Computational Physics, National Sun Yat-sen University, Kaohsiung 80424, Taiwan (R.O.C.); ∥ Physics Division, National Center for Theoretical Sciences, Taipei 10617, Taiwan (R.O.C.)

## Abstract

A facile synthesis of a rigid fully sp^2^-hybridized spheriphane C_48_H_30_ is reported. Starting from isophthalaldehyde and 2’-bromoacetophenone, a precursor containing six aldehyde groups was synthesized in four steps, which was further assembled into the spheriphane structure by three intramolecular McMurry reactions. Due to the orthogonally arranged alkenes and *o*-phenylene moieties, this spheriphane has the potential to be developed as a novel building block for framework materials.

## Graphical abstract

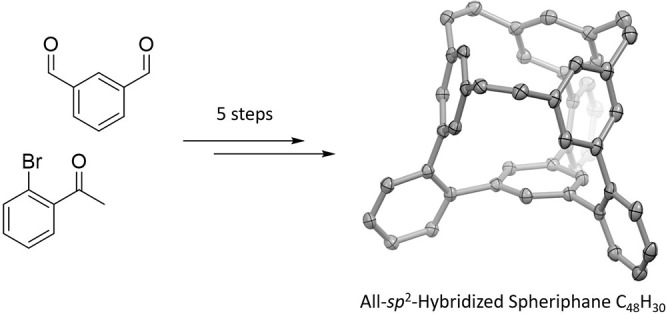

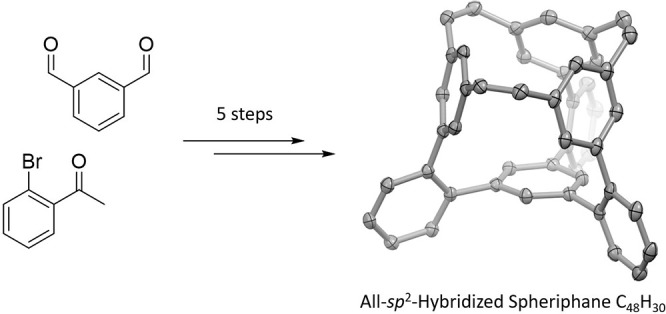

Highly crowded 3-dimensional polycyclic aromatic molecules have attracted increasing attention in recent years. The interest in these kinds of molecules can be viewed as a continuous pursuit toward the still elusive porous 3-dimensional carbon allotropes, such as cubic graphite^1^ or carbon schwarzites.^2^ These fully *sp* ^2^-hybridized carbon allotropes were predicted to have intriguing properties and may find applications in spintronics and energy storage.^3^

The studies of spherical cyclophanes, or spheriphanes, represent an early example of the investigation of highly crowded 3-dimensional polycyclic molecules. Such work is pioneered by Vögtle and co-workers, and a family of spheriphanes with aromatic rings linked by *sp* ^3^-hybridized carbon or heteroatom linkers was reported.^4^ In particular, the highly symmetric parent spheriphane **1** represents an interesting candidate for π extension. (Figure 1) This idea was tested by Kuck and co-workers two decades ago,^5^ and triple benzannulated spheriphane **2** with three saturated ethylene bridges was synthesized. The still elusive sextuple benzannulated spheriphane **3** was also proposed as a target by functionalization of **2**, in which the highly symmetric **3** also resembles the 3-dimensional centrohexaindane developed by Kuck.^6^ However, to the best of our knowledge, no further reports on related compounds have been published except for a patent.^7^ Here, we report the synthesis of spheriphane **4**, a fully *sp* ^2^-hybridized version of **2** with 1,2-ethenylide bridges, in five steps from commercially available chemicals.

**Figure 1:**
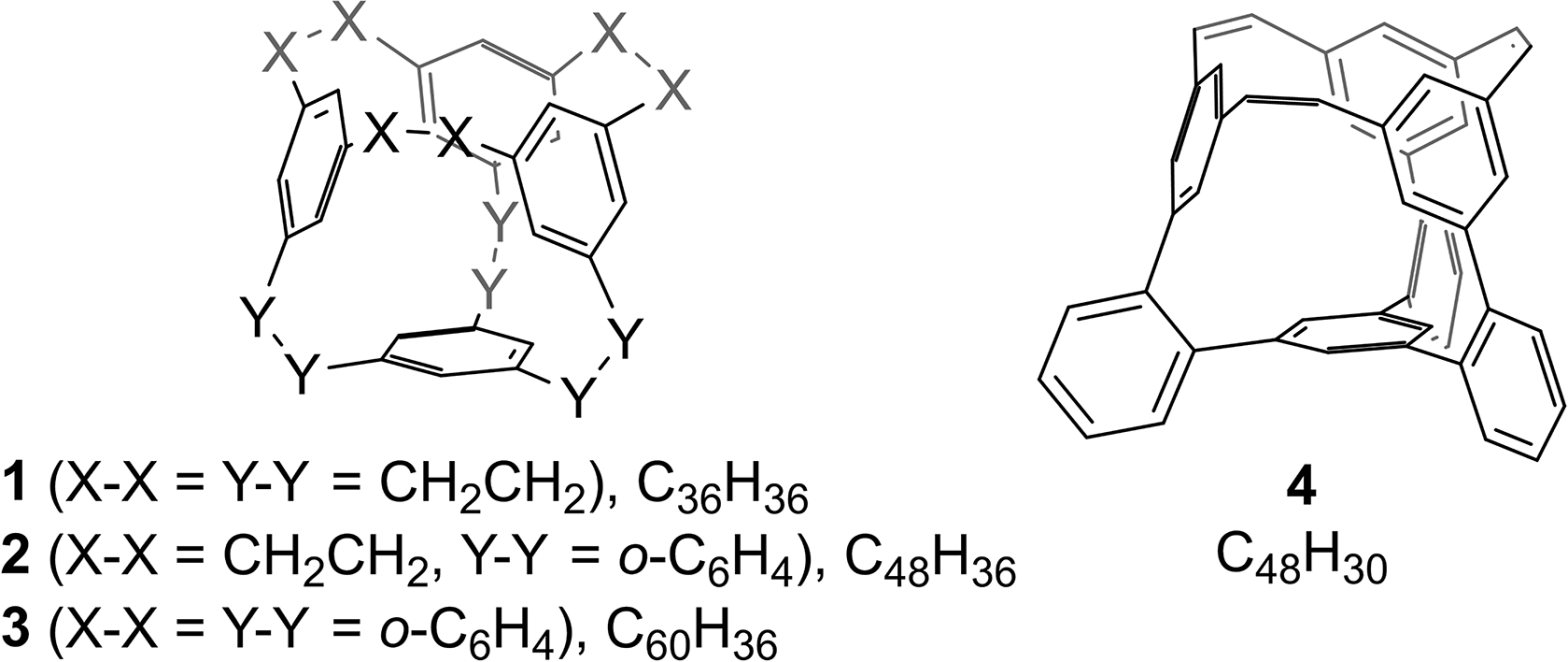
Structures of reported spheriphane **1** and **2**; hypothetical spheriphane **3**; and spheriphane **4** presented in this work.

The synthesis of spheriphane **4** is shown in Scheme 1. Isophthalaldehyde was brominated to give **6**, which was further borylated with Miyaura borylation to yield the boronic ester **7**.^8^

**Scheme 1:**
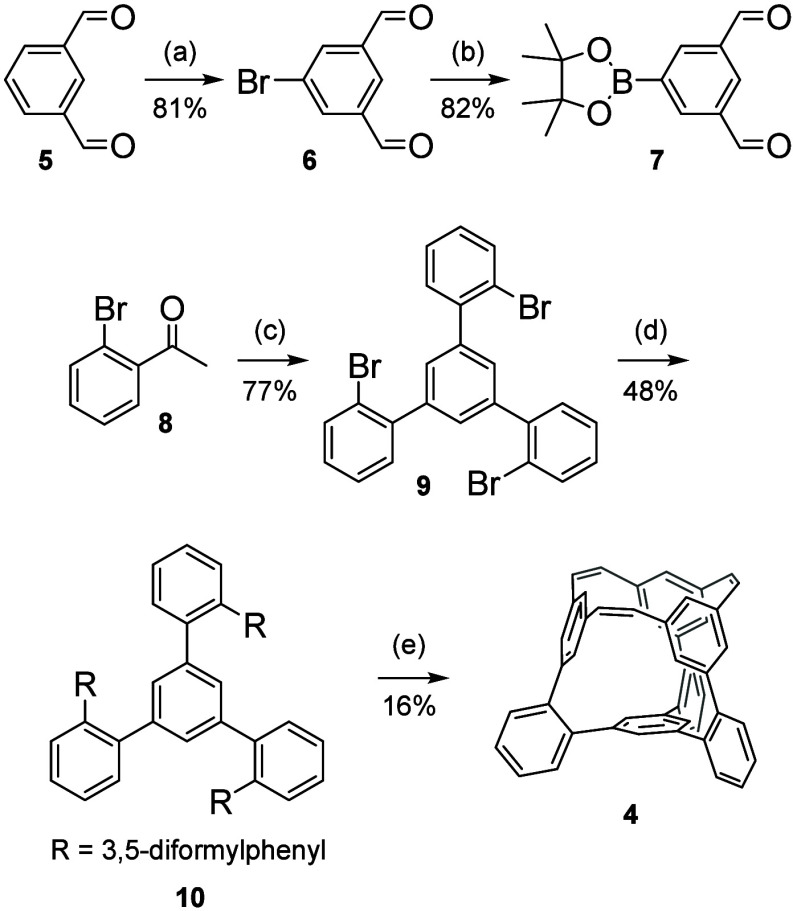
Synthesis of Spheriphane **4** ^a^

a Reagents and conditions: (a) *N*-bromosuccinimide, H_2_SO_4_, 65 °C; (b) bis­(pinacolato)­diboron, KOAc, Pd­(dppf)­Cl_2_, 1,4-dioxane, 90 °C; (c) CF_3_SO_3_H, 130 °C; (d) **7**, Pd­(PPh_3_)_2_Cl_2_, K_2_CO_3_, 1,4-dioxane/H_2_O, 85 °C; (e) Zn powder, Cp_2_TiCl_2_, 1,4-dioxane, 100 °C.

2’-bromoacetophenone was trimerized with triflic acid to give compound **9**,^9^ which was coupled with compound **7** in a Suzuki reaction to yield compound **10**. The symmetric NMR spectra of **10** indicated that it exists as a dynamic equilibrium between different conformers at ambient temperature, similar to structurally related compounds.^5^

Conventional McMurry reaction conditions on compound **10** were attempted using titanium tetrachloride as the titanium source and zinc powder as the reducing agent.^10^ However, only complex mixtures were obtained without the formation of the target spheriphane **4**. When titanocene dichloride was used as the titanium source,^11^ spheriphane **4** was obtained in 16% yield after purification. The different results obtained using different titanium sources are worth noting. Not-well-defined “low-valent titanium” species of Ti(0) or Ti­(II) are generally accepted as the active species in the McMurry reaction when titanium chlorides are used as the titanium source.^10^ On the other hand, a relatively well-defined Ti­(III) species, the Nugent’s reagent, was proposed as the active species by using titanocene dichloride as the titanium source.^11^ In addition, titanocene dichloride is a relatively stable crystalline compound that can be easily handled, while samples of titanium chlorides may vary in quality and can lead to extra complexity in reproducibility and yield. Such differences between the two reagents may contribute to the observed outcomes when performing the McMurry reaction on compound **10**.

Compound **4** was first characterized by NMR spectroscopy. The highly symmetric proton NMR spectrum of **4** clearly indicates a C_3v_ molecular symmetry, and the peaks were assigned to the corresponding protons shown in Figure 2 based on its NOESY NMR spectrum (Figure S1).

**Figure 2:**
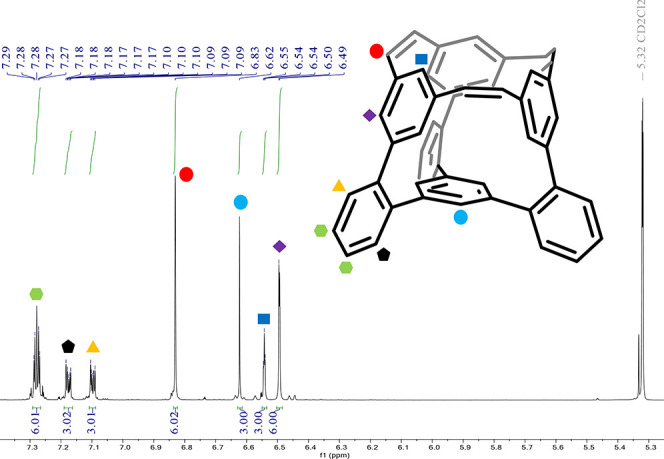
Proton NMR spectrum of spheriphane **4** in CD_2_Cl_2_ with the assignment of the peaks.

Spheriphane **4** dissolves in organic solvents to form colorless solutions. Figure 3 shows the absorption and emission spectra of **4** in 1,4-dioxane. The absorption spectrum shows a single peak at 219 nm. When excited at 219 nm, **4** shows an emission spectrum with a peak at 288 nm. This is in contrast to the previously reported **2**, in which three absorption peaks at 261 nm, 220 and 216 nm were reported.^5^ This blue-shifted longest wavelength absorption of **4** compared to **2** indicates that the unsaturated bridges cannot effectively lead to an extended conjugation.

**Figure 3:**
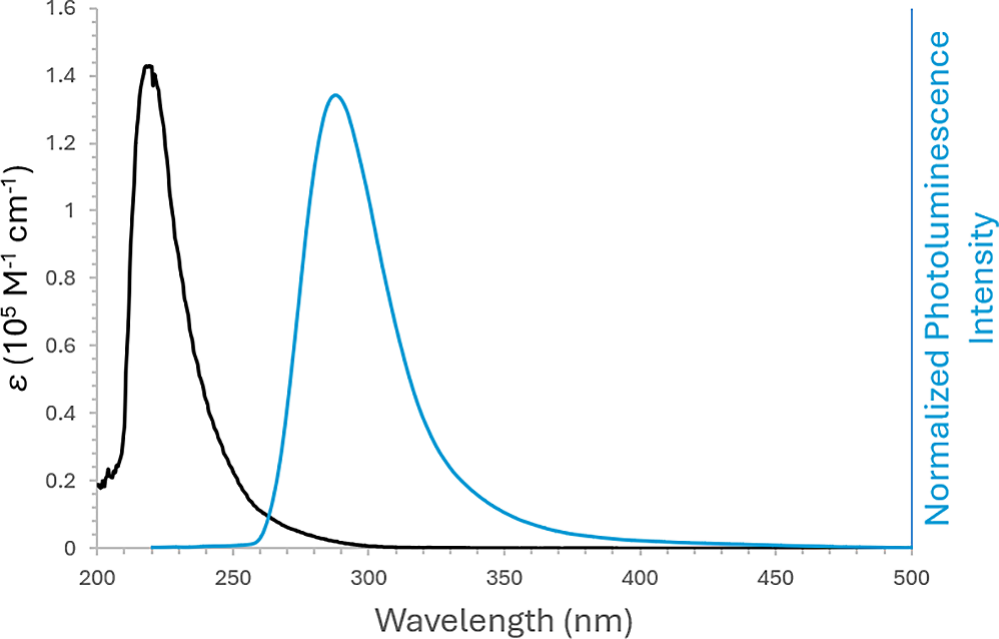
Absorption and normalized emission spectra (excited at 219 nm) of spheriphane **4** in 1,4-dioxane (1 × 10^–5^ M).

Colorless crystals suitable for X-ray crystallography were obtained by slow diffusion of methanol vapor into a solution of **4** in toluene/dichloromethane. The solid-state structure of **4** slightly deviates from the perfect C_3v_ symmetry (Figure 4). All bond lengths are in the typical range.^12^ The alkene double bonds are 1.33Å long, all *sp* ^2^-*sp* ^2^ carbon–carbon single bonds connecting the aromatic rings and the alkene moieties are 1.49–1.50Å long, and the bonds within aromatic rings are 1.38–1.40Å long. As a subunit of the highly symmetric spheriphane **3**, which can also be viewed as topologically similar to centropolyindanes,^6,13^ the *o*-phenylene moieties and alkene moieties are pointing away from the centroid of the spheriphane cavity roughly along the x, y, and z axes of the Cartesian coordinate system (Figures S2–S4). With such a symmetric topology and rigid *sp* ^2^-hybridized framework, **4** might serve as a novel building block for 3-dimensional framework materials.^14^

**Figure 4:**
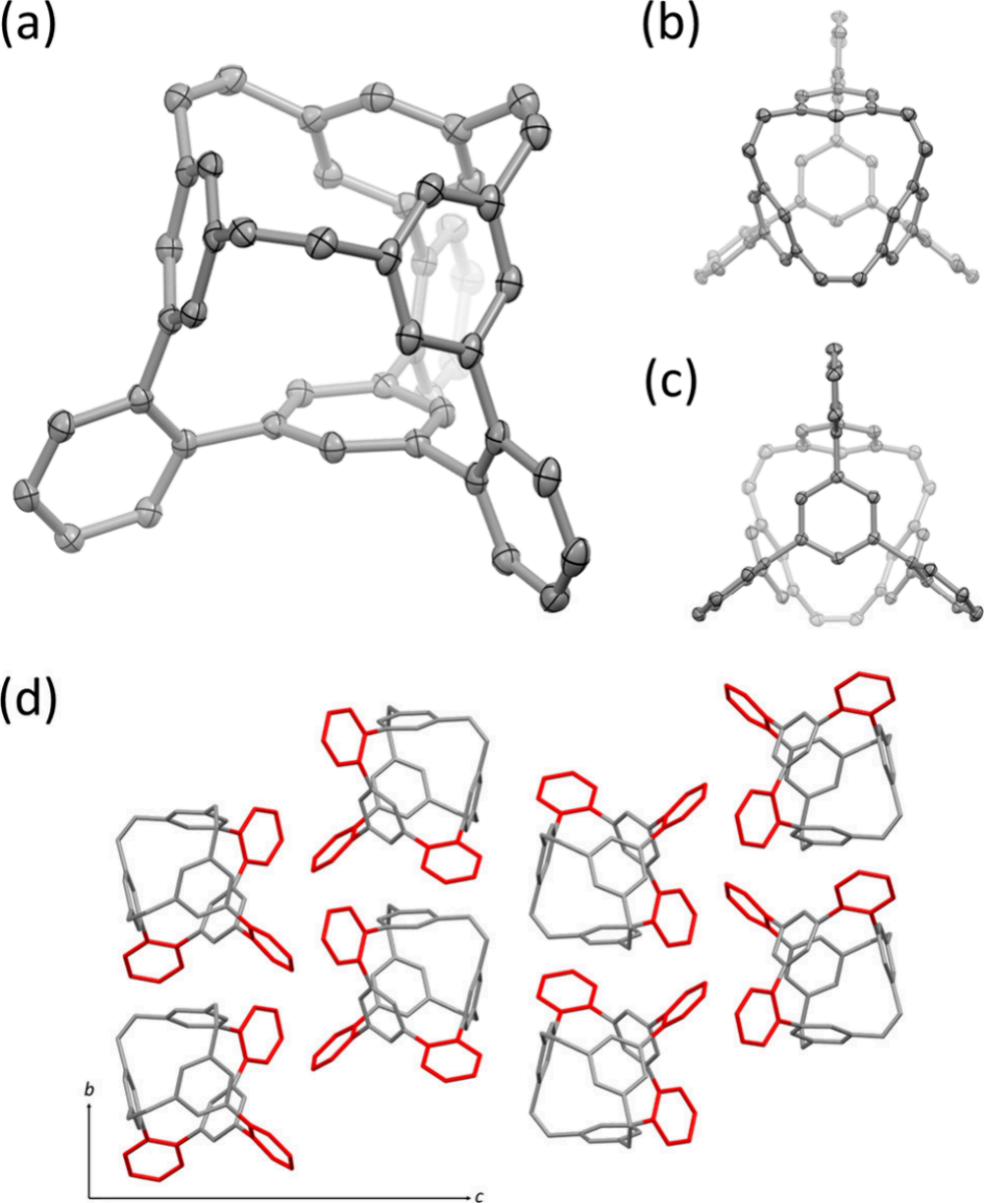
Crystal structure of **4**. (a) side view; (b) top view; (c) bottom view; (d) packing structure of **4** viewing along the *a* axis, with the *o*-phenylene moieties highlighted in red. (Hydrogen atoms removed for clarity and carbon atom positions shown as ellipsoids at 50% probability level for (a), (b), and (c).).

The cavity within **4** was analyzed by the MoloVol program^15^ based on the crystal data of **4**. When using a hydrogen atom as the probe (radius 1.20Å), no cavity was detected, which implies that the cavity in **4** is smaller than the van der Waals radius of a hydrogen atom. By varying the size of the probe, the largest spherical probe that can fit into the cavity of **4** has a radius 1.12Å; while a probe with a radius of 0.42 Å can pass through the openings freely into the cavity (refer to the Supporting Information for details).

Spheriphane **4** and related compounds were further analyzed by quantum chemical calculations. The molecular orbitals of **4** and **2** are shown in Figure 5. Both compounds show (within the accuracy of the calculations) triply degenerate HOMO and doubly degenerate LUMO. In contrast, spheriphane **1** only shows a doubly degenerate HOMO (−5.92 eV) and a nondegenerate LUMO (−0.03 eV) (Figure S7). Both **4** and **2** have their HOMOs mainly distributed over the four phenine moieties that form the cage structures, while contributions from the *o*-phenylene moieties are evident in their LUMOs. There is no obvious contribution from the π orbitals of the 1,2-ethenylide bridges of **4** to its frontier molecular orbitals. The frontier molecular orbitals of **4** are slightly lower in energy than those of **2**.

**Figure 5:**
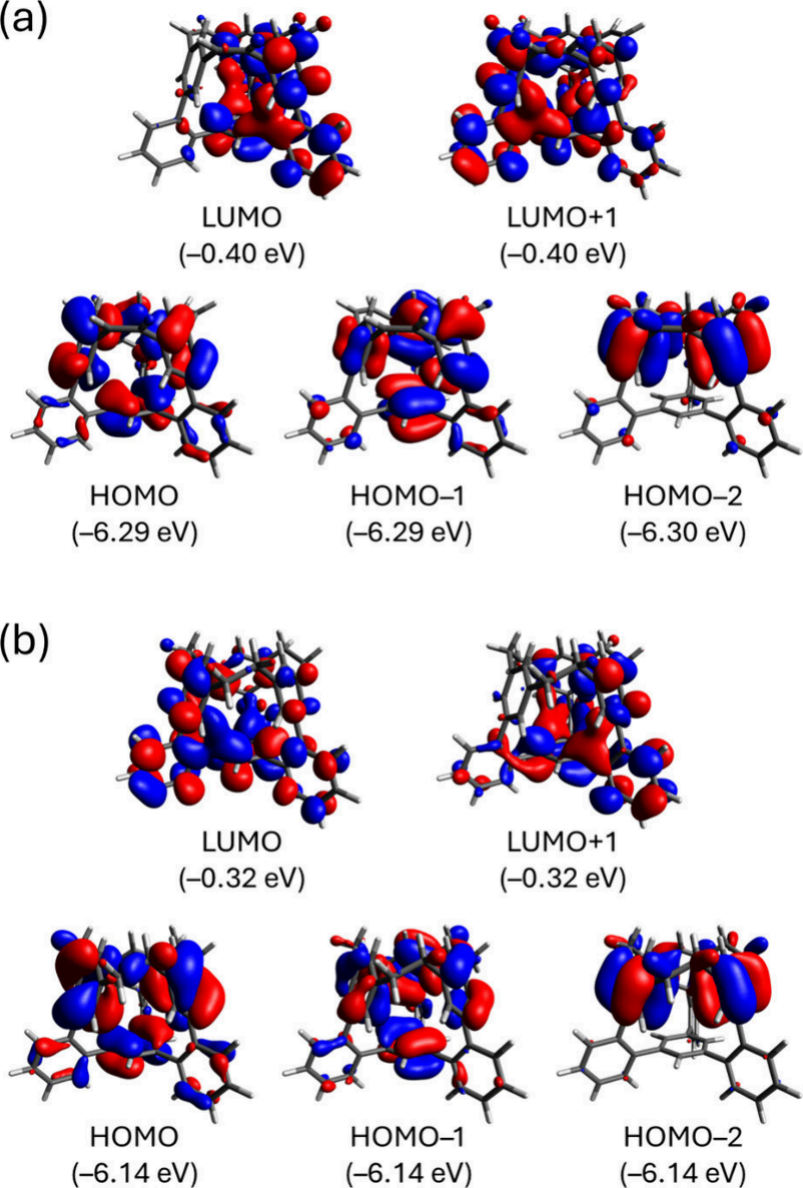
Molecular orbitals of (a) **4** and (b) **2** calculated at the B3LYP/6–31G­(d) level of theory (isovalue: 0.02).

Nucleus-Independent Chemical Shifts (NICS)^16^ calculations were performed on individual rings and at the center of the cage cavity, defined by the centroid of the four phenine rings, to assess the aromaticity of the molecules. As shown in Figure 6, the NICS(0) values of different types of rings in spheriphane **1**, **2**, and **4** are in the typical range for aromatic rings, indicating usual aromatic properties for these rings. Harmonic oscillator model of aromaticity (HOMA)^17^ analysis also gives similar results (Figure S8). The NICS values at the cavity center of all three spheriphanes show values comparable to other aromatic rings, indicating that these cavity centers are strongly magnetically shielded. This is in line with the cage cavities being located at the shielding region of the four phenine rings. An interesting trend in the NICS values at the cavity center can also be observed: This value is the highest for spheriphane **1** and the lowest for spheriphane **4**. This is consistent with the increasing number of *o*-phenylene rings and 1,2-ethenylide bridges surrounding the cavity by comparing the structures of **1**, **2**, and **4**, in which both *o*-phenylene rings and 1,2-ethenylide bridges will have their deshielding region covering the cavity center.

**Figure 6:**
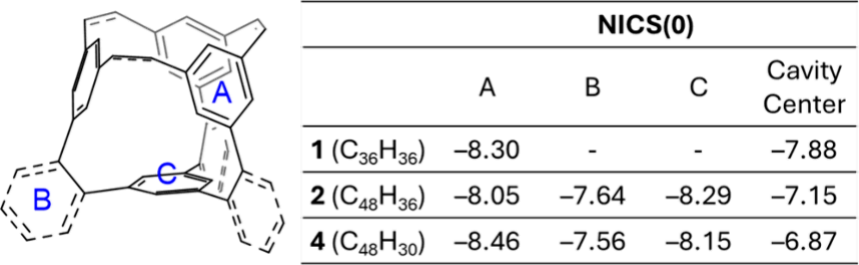
NICS­(0) values at different locations for **1**, **2**, and **4** calculated at the B3LYP/6–311+G­(2d,p)//B3LYP/6–31G­(d) level of theory.

In summary, we have successfully synthesized an all-*sp* ^2^-hybridized spheriphane **4** in five steps from commercially available chemicals, and its structure is unambiguously characterized by X-ray crystallography. Further benzannulation at the alkene bridges can potentially lead to the highly symmetrical spheriphane **3** with *T*_d_ symmetry. Such structures will have great potential to act as building blocks for framework materials by taking advantage of the six *o*-phenylene rings pointing orthogonally in the Cartesian coordinate system. Further studies on realizing such ideas are in progress in our laboratory.

## Supplementary Material









## Data Availability

The data underlying this study are available in the published article and its .

## Notes

Text file of all computed molecule Cartesian coordinates in a format for convenient visualization (XYZ)
Text file of all computed molecule Cartesian coordinates in a format for convenient visualization (XYZ)
Text file of all computed molecule Cartesian coordinates in a format for convenient visualization (XYZ)
Details of synthesis and characterization, NMR spectra, theoretical calculations, and crystallographic information for **4** (PDF)

## References

[1] Gibson J., Holohan M., Riley H. L. (1946). 87. Amorphous Carbon. J. Chem. Soc..

[2] Mackay A. L., Terrones H. (1991). Diamond from Graphite. Nature.

[3] Baughman R. H., Cui C. (1993). Polymers with Conjugated
Chains in
Three Dimensions. Synth. Met..

[4] Vögtle F., Gross J., Seel C., Nieger M. (1992). C _36_ H _36_ —Tetrahedral
Clamping of Four Benzene Rings in a Spherical Hydrocarbon Framework. Angew. Chem., Int. Ed. Engl..

[5] Nierle J., Kuck D. (2006). Synthesis of a Benzoannelated Spheriphane
(Globular Cyclophane) on
the Way to Potential Precursors of C _60_ -Fullerene. Synlett.

[6] Kuck D. (2015). From Fragmentation
to Construction—from Void to Massive: Fascination with Organic
Mass Spectrometry and the Synthesis of Novel Three-Dimensional Polycyclic
Aromatic Hydrocarbons. Chem. Rec..

[7] Johnson, D. W. ; Collins, M. Synthesis of Cyclophanes from a Self-Assembly Reaction. US20160137598A1, 2016.

[8] Mondal B., Ghosh A. K., Mukherjee P. S. (2017). Reversible
Multistimuli Switching
of a Spiropyran-Functionalized Organic Cage in Solid and Solution. J. Org. Chem..

[9] Cui S., Zhuang G., Lu D., Huang Q., Jia H., Wang Y., Yang S., Du P. (2018). A Three-Dimensional
Capsule-like Carbon Nanocage as a Segment Model of Capped Zigzag [12,0]
Carbon Nanotubes: Synthesis, Characterization, and Complexation with
C _70_. Angew. Chem., Int. Ed..

[10] McMurry J. E. (1989). Carbonyl-Coupling Reactions Using
Low-Valent Titanium. Chem. Rev..

[11] Diéguez H. R., López A., Domingo V., Arteaga J. F., Dobado J. A., Herrador M. M., Quílez Del Moral J. F., Barrero A. F. (2010). Weakening
C–O Bonds: Ti­(III), a New Reagent for Alcohol Deoxygenation
and Carbonyl Coupling Olefination. J. Am. Chem.
Soc..

[12] Anslyn, E. V. ; Dougherty, D. A. Modern Physical Organic Chemistry; University Science: Sausalito, CA, 2004; Chapter 1, page 22.

[13] Kuck D. (2006). Functionalized
Aromatics Aligned with the Three Cartesian Axes: Extension of Centropolyindane
Chemistry. Pure Appl. Chem..

[14] Guan X., Chen F., Fang Q., Qiu S. (2020). Design and Applications
of Three Dimensional Covalent Organic Frameworks. Chem. Soc. Rev..

[15] Maglic J. B., Lavendomme R. (2022). *MoloVol*: An Easy-to-Use Program for
Analyzing Cavities, Volumes and Surface Areas of Chemical Structures. J. Appl. Crystallogr..

[16] Schleyer P.
v R., Maerker C., Dransfeld A., Jiao H., van Eikema Hommes N.
J. R. (1996). Nucleus-Independent
Chemical Shifts: A Simple and Efficient Aromaticity Probe. J. Am. Chem. Soc..

[17] Kruszewski J., Krygowski T. M. (1972). Definition
of Aromaticity Basing on the Harmonic Oscillator Model. Tetrahedron Lett..

