# Supplementary material for: Synthesis of an All-sp2‑Hybridized Spheriphane C48H30
Source: Org Lett. 2026 Jun 8;28(24):7918–22. doi: 10.1021/acs.orglett.6c02209 (PMC13288921; doi:10.1021/acs.orglett.6c02209)
Supplement: Supplementary file 4 [file ol6c02209_si_004.pdf]

Supporting Information

Synthesis of an All-sp^2-Hybridized Spheriphane $C_{48}H_{30}$

Jing-Yuan Wang[1], Chang-Rui Chen[1], Che-Wei Chang[1], Cheng-chau Chiu[1,2,3,4] and Kwan Yin Cheung*[1]

[1]*Department of Chemistry, National Sun Yat-sen University, Kaohsiung 80424, Taiwan (R.O.C.)*

[2]*Green Hydrogen Research Center, National Sun Yat-sen University, Kaohsiung 80424, Taiwan (R.O.C.)*

[3]*Center for Theoretical and Computational Physics, National Sun Yat-sen University, Kaohsiung 80424, Taiwan (R.O.C.)*

[4]*Physics Division, National Center for Theoretical Sciences, Taipei 10617, Taiwan (R.O.C.)*

E-mail: davidcky@mail.nsysu.edu.tw

Contents

1. Materials and Methods........2

2. Synthesis of Compounds........3

3. Crystal Structures and Crystal Data........9

4. Cavity analysis of 4........12

5. Quantum Chemical Calculations........14

6. Harmonic Oscillator Model of Aromaticity (HOMA) Analysis........15

7. Reference........16

8. NMR Spectra........18

1. **Materials and Methods**

The reagents and starting materials employed were commercially available and used without any further purification unless otherwise noted or made following reported methods as indicated. Unless stated otherwise, the reaction mixtures were heated by placing the reaction flasks in an oil bath at the indicated temperature, as measured by a temperature probe. Analytical thin layer chromatography (TLC) was done using silica Gel (E. Merck silica gel 60 F254 precoated plates). Flash column chromatography was performed using silica gel (300-400 mesh). Chromatograms were visualized with UV light (254 and 365 nm). Anhydrous and O_2-free solvents were purified by a Vigor VSPS-5 solvent purification system. Preparative recycling gel permeation chromatography (GPC) was performed with a JAI LaboACE LC-5060 Plus II instrument equipped with JAIGEL-2HR Plus columns using dichloromethane as the eluent. Nuclear magnetic resonance (NMR) spectra were recorded on Bruker Avance III 300 (^{1}H 300 MHz, ^{13}C 75 MHz), JEOL ECZS 400 (^{1}H 400 MHz, ^{13}C 100 MHz) and JEOL ECZ 600R (^{1}H 600 MHz, ^{13}C 150 MHz) spectrometers. Chemical shifts (δ) were given in parts per million (ppm) and referenced to $CDCl_3$ (^{1}H: 7.26 ppm; ^{13}C:77.16 ppm) or CD_2Cl_2 (^{1}H: 5.32 ppm). Coupling constants (*J*) were given in Hertz (Hz) and referred to apparent multiplicities (s = singlet, d = doublet, t = triplet, q = quartet, m = multiplet, dd = doublet of doublets, *etc*.). UV-vis absorption spectrum was recorded on a METTLER TOLEDO EasyPlus UV/VIS spectrophotometer. Photoluminescence spectrum was taken on a JASCO FP-8650 spectrofluorometer. High resolution mass spectra (HRMS) were collected on Bruker ultrafleXtreme (MALDI-TOF) (Laser type: NdYAG 355 nm) by the Instrument Center at National Chung Hsing University. Single-crystal X-ray crystallography data were collected on a Rigaku XtaLAB Synergy DW diffractometer by the Instrument Center at National Tsing Hua University.

2. **Synthesis of Compounds**

5-bromoisophthalaldehyde (6)

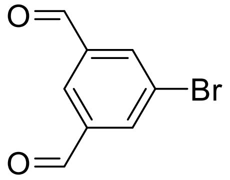

Isophthalaldehyde (0.27 g, 2.0 mmol, 1.0 equiv.) and concentrated sulfuric acid (1.1 mL, 20 mmol, 10 equiv.) were added to a reaction flask and the mixture was stirred at 65 °C. *N*-bromosuccinimide (0.39 g, 2.2 mmol, 1.1 equiv.) was then added slowly, and the reaction was maintained at the same temperature for 24 h. After cooling to room temperature, ice water was added, resulting in the precipitation of a solid. The solid was collected by filtration and extracted with deionized water and dichloromethane. The organic layer was dried over anhydrous Na_2SO_4, filtered, and was concentrated with a rotary evaporator. The crude product was purified by filtering through a silica gel plug with dichloromethane as eluent to afford the product **6** as a beige solid (0.35 g, 81 %).

^{1}H NMR (300 MHz, $CDCl_3$) δ 10.05 (s, 2H), 8.30 (t, J = 1.43 Hz, 1H), 8.26 (d, J = 1.43 Hz, 2H).

^{13}C NMR (100 MHz, $CDCl_3$) δ 189.6, 158.5, 137.3, 129.3, 124.5.

The NMR spectra are consistent with the reported.[1]

Compound 7

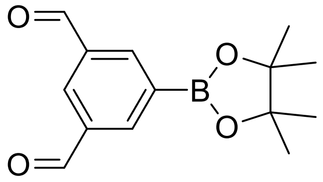

5-bromoisophthalaldehyde (**6**) (0.22 g, 0.85 mmol, 1.0 equiv.), bis(pinacolato)diboron (0.38 g, 1.5 mmol, 1.4 equiv.), potassium acetate (0.29 g, 3.0 mmol, 2.9 equiv.), and [1,1'-bis(diphenylphosphino)ferrocene]dichloropalladium(II) (36.6 mg, 0.05 mmol, 5 mol%) were added to a reaction flask, which was degassed and charged with 1,4-dioxane (10 mL). The mixture was further bubbled with nitrogen for 10 minutes and the flask was sealed and stirred at 90 °C for 12 h. After completion, the reaction mixture was extracted with deionized water and EtOAc. The organic layer was dried over anhydrous Na_2SO_4, filtered, and concentrated with a rotary evaporator. The crude product was purified by column chromatography (hexane/EtOAc = 1/1) to give the product **7** as a white solid (0.22 g, 82 %).

^{1}H NMR (300 MHz, $CDCl_3$) δ 10.13 (s, 1H), 8.55 (d, *J* = 1.73 Hz, 2H), 8.45 (t, *J* = 1.73 Hz, 1H), 1.38 (s, 12H).

^{13}C NMR (100 MHz, $CDCl_3$) δ 191.3, 141.4, 136.4, 132.2, 84.8, 24.9. (boron alpha carbon not visible)

The NMR spectrum is consistent with the reported.[1]

1,3,5-tri(2-bromophenyl)benzene (9)

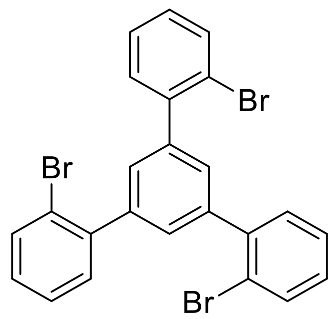

2'-bromoacetophenone (0.90 mL, 6.7 mmol, 1 equiv.) and trifluoromethanesulfonic acid (0.09 mL, 1.0 mmol, 0.15 equiv.) were added to a reaction flask. The mixture was stirred at 130 °C for 12 h. After cooling to room temperature, the reaction mixture was quenched with deionized water and extracted with dichloromethane. The organic layer was dried over anhydrous Na_2SO_4, filtered, and concentrated with a rotary evaporator. The crude product was purified by column chromatography (hexane/dichloromethane = 1/1) to afford the product **9** as a white solid (2.80 g, 77 %).

^{1}H NMR (400 MHz, $CDCl_3$) δ 7.70 (dd, *J* = 8.00, 1.30 Hz, 3H), 7.51 (s, 3H), 7.47 (dd, *J* = 7.70, 1.70 Hz, 3H), 7.38 (td, *J* = 7.50, 1.30 Hz, 3H), 7.22 (td, *J* = 8.00, 1.80 Hz, 3H).

^{13}C NMR (100 MHz, $CDCl_3$) δ 142.1, 140.5, 133.3, 131.6, 129.7, 129.0, 127.5, 122.8.

The NMR spectra are consistent with the reported.[2]

Compound 10

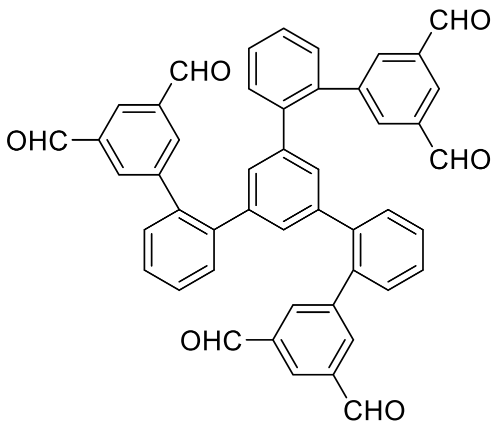

9 (2.35 g, 4.32 mmol, 1.0 equiv.), **7** (3.93 g, 15.1 mmol, 3.5 equiv.), bis(triphenylphosphine)palladium(II) dichloride (154.4 mg, 0.22 mmol, 5 mol%), and K_2CO_3 (4.18 g, 30.24 mmol, 7.0 equiv.) were added to a reaction flask, which was degassed and charged with 1,4-dioxane/H_2O (5/1, 60 mL). The reaction mixture was further bubbled with nitrogen for 10 minutes and the flask was sealed and stirred at 85 °C for 16 h. After completion, the reaction mixture was extracted with deionized water and EtOAc. The organic layer was dried over anhydrous Na_2SO_4, filtered, and concentrated with a rotary evaporator. The crude product was purified by column chromatography (hexane/EtOAc = 3/1), followed by gel permeation chromatography (GPC) to afford the product **10** as a light yellow solid (1.46 g, 48 %).

mp: 149-155 °C

^{1}H NMR (400 MHz, $CDCl_3$) δ 9.98 (s, 6H), 8.21 (s, 3H), 7.84 (d, J = 1.52 Hz, 6H), 7.43-7.39 (m, 3H), 7.36-7.32 (m, 6H), 6.84 (d, J = 6.76 Hz, 3H), 6.81 (s, 3H).

^{13}C NMR (100 MHz, $CDCl_3$) δ 190.9, 143.5, 140.4, 139.4, 137.3, 136.8, 135.5, 130.6, 130.4, 130.1, 129.1, 128.7, 128.2.

HRMS (MALDI-TOF) calcd. for $C_{48}H_{30}O_6Na$ ($[M+Na]^+$): 725.1940, found 725.1927.

Compound 4

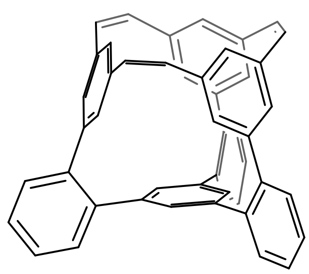

Under a nitrogen atmosphere, a Schlenk flask was charged with Zn powder (0.28 g, 4.269 mmol, 30.0 equiv.), Cp_2TiCl_2 (0.53 g, 2.314 mmol, 15.0 equiv.), and anhydrous dioxane (25 mL). The mixture was heated at reflux (100 °C) for 2.5 h. After cooling to room temperature, a solution of the carbonyl compound **10** (100 mg, 0.142 mmol, 1.0 equiv.) in anhydrous dioxane (100 mL) was added slowly by a syringe. Following the addition, the reaction mixture was heated at 100 °C for an additional 12 h. The reaction was then quenched with an aqueous 10% K_2CO_3 solution and extracted with dichloromethane. The organic layer was dried over anhydrous Na_2SO_4, filtered, and concentrated with a rotary evaporator. The crude product was purified by column chromatography (hexane/dichloromethane = 2/1), followed by gel permeation chromatography (GPC), to afford the product **4** as a white solid (13.8 mg, 16 % yield).

mp: no melting was observed upon heating up to 400 °C

^{1}H NMR (600 MHz, CD_2Cl_2) δ 7.29-7.27 (m, 6H), 7.18-7.17 (m, 3H), 7.10-7.09 (m, 3H), 6.83 (s, 6H), 6.62 (s, 3H), 6.54 (t, J = 1.8 Hz, 3H), 6.50 (d, J = 1.56 Hz, 6H).

^{13}C NMR (150 MHz, $CDCl_3$) δ 142.0, 141.9, 141.4, 140.7, 137.4, 133.8, 129.6, 129.1, 128.2, 127.5, 127.2, 126.5, 126.5.

HRMS (MALDI-TOF) calcd. for $C_{48}H_{30}$ ($[M]^+$): 606.2348, found 606.2346.

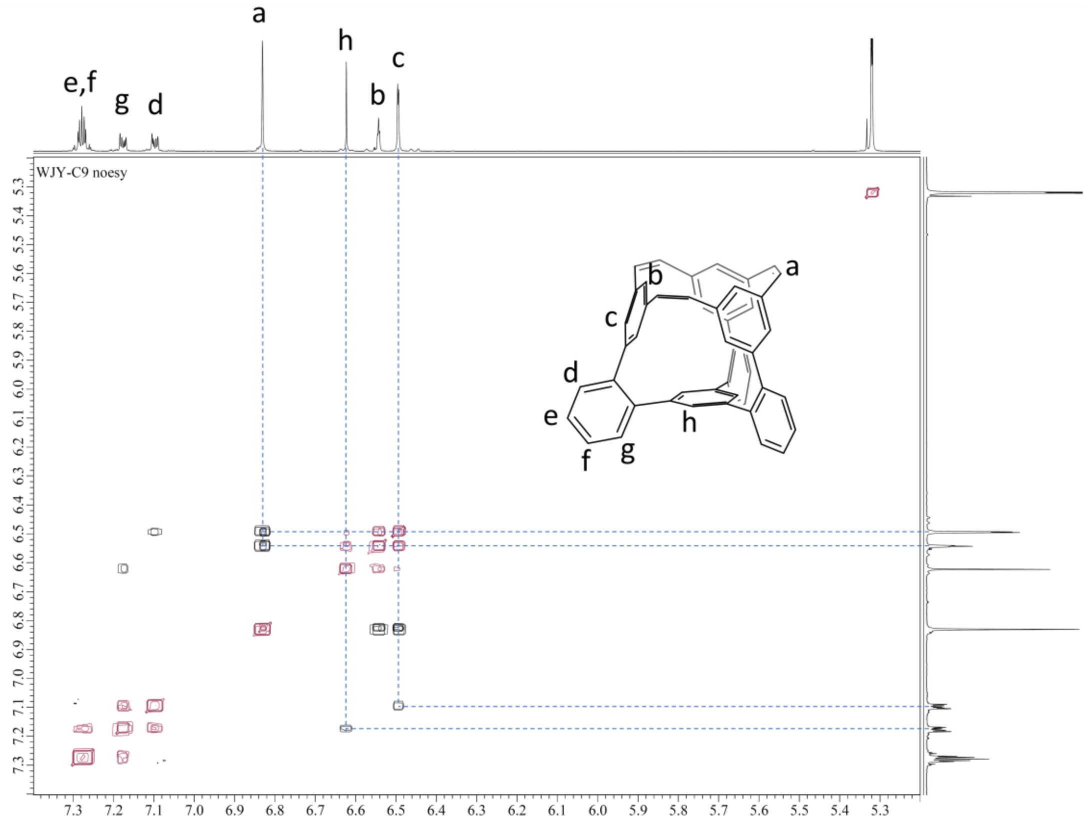

Figure S1. NOESY ^{1}H NMR spectrum of **4** (600 MHz, CD_2Cl_2).

3. **Crystal Structures and Crystal Data**

X-ray crystallography data were collected on a Rigaku XtaLAB Synergy DW diffractometer.

Table S2. Crystallographic Data of **4**

Empirical formula	$C_{48}H_{30}$
Formula weight	606.72
Temperature/K	100.00(16)
Crystal system	monoclinic
Space group	$P2_1/c$
a/Å	9.44730(10)
b/Å	9.06870(10)
c/Å	37.9245(4)
α/°	90
β/°	92.6350(10)
γ/°	90
Volume/Å^3	3245.74(6)
Z	4
ρ_{calc}g/cm^3	1.242
μ/mm^{-1}	0.534
F(000)	1272.0
Crystal size/mm^3	0.15 × 0.13 × 0.04
Radiation	Cu Kα (λ = 1.54184)
2Θ range for data collection/°	9.338 to 145.572
Index ranges	$-11 \le h \le 11$, $-7 \le k \le 10$, $-46 \le l \le 46$
Reflections collected	52706
Independent reflections	6355 [R_{int} = 0.0292, R_{sigma} = 0.0146]
Data/restraints/parameters	6355/0/434
Goodness-of-fit on F^2	1.049
Final R indexes [I>=2σ (I)]	R_1 = 0.0372, wR_2 = 0.0919
Final R indexes [all data]	R_1 = 0.0404, wR_2 = 0.0937
Largest diff. peak/hole / e Å^{-3}	0.22/-0.19

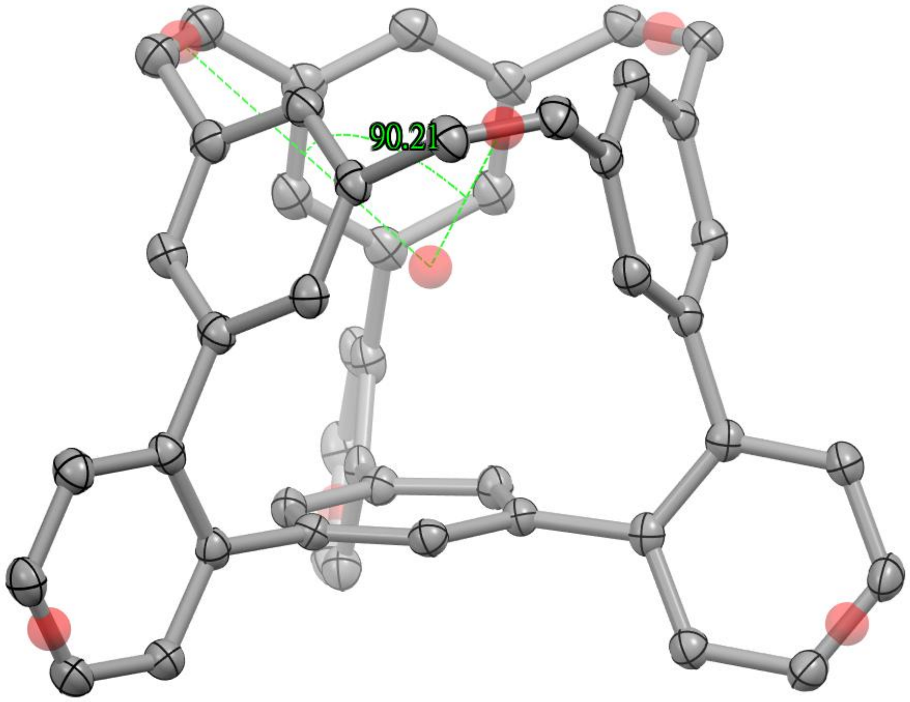

Figure S2. Angle between two alkene moieties with respect to the centroid of the four phenine rings in the crystal structure of **4**.

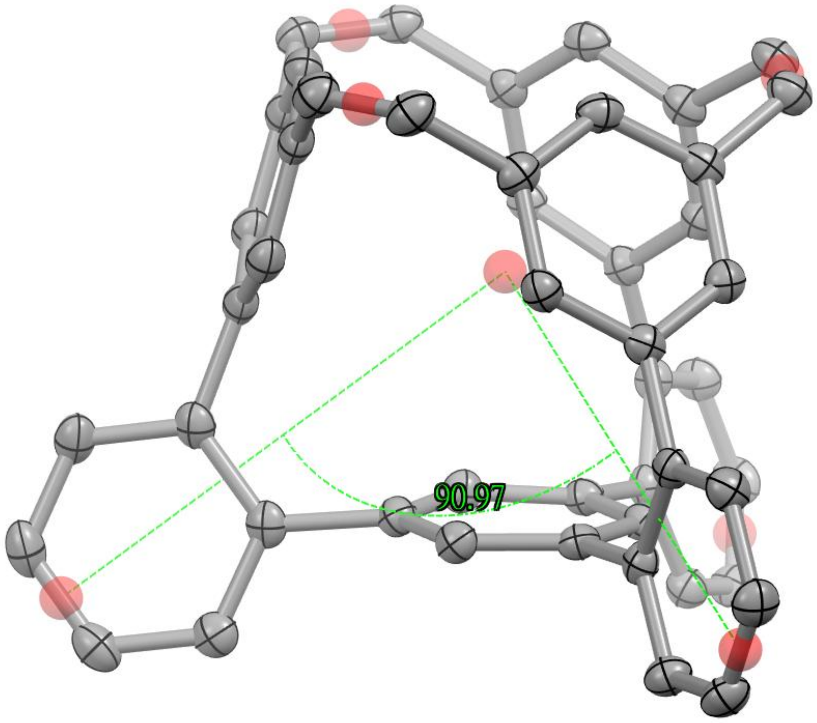

Figure S3. Angle between two *o*-phenylene moieties with respect to the centroid of the four phenine rings in the crystal structure of **4**.

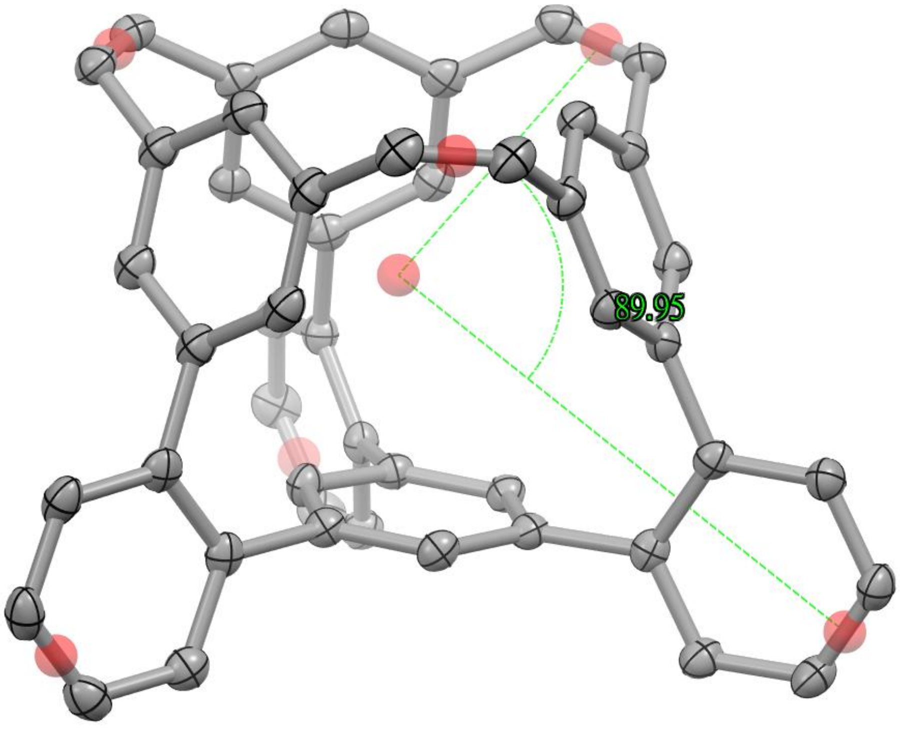

Figure S4. Angle between an alkene moiety and an *o*-phenylene moiety with respect to the centroid of the four phenine rings in the crystal structure of **4**.

4. **Cavity analysis of 4**

The cavity within **4** was analyzed with the MoloVol program[3] (version 1.2.0) based on the crystal data of **4**.

The following parameters were applied:

The default element file provided by the MoloVol program was used. The CIF file of **4** was used as the input. The single-probe mode was used with a Grid resolution of 0.1Å and an Optimization depth of 4 was used. The probe radius value was varied to estimate the cavity size and the size of the openings of the structure.

An isolated cavity within the structure of **4** was detected by the program when the probe had a radius ranging from 0.43Å to 1.12Å. This implies that a probe with a radius larger than this range cannot fit into the cavity of **4**, while a probe with a radius smaller than this range can move freely into the cavity of **4** through the openings of the structure.

Since the MoloVol program treats atoms and probes as static impenetrable spheres, the result obtained here is only a rough estimation of the potential size of guest species that can bind to the cavity of **4**.

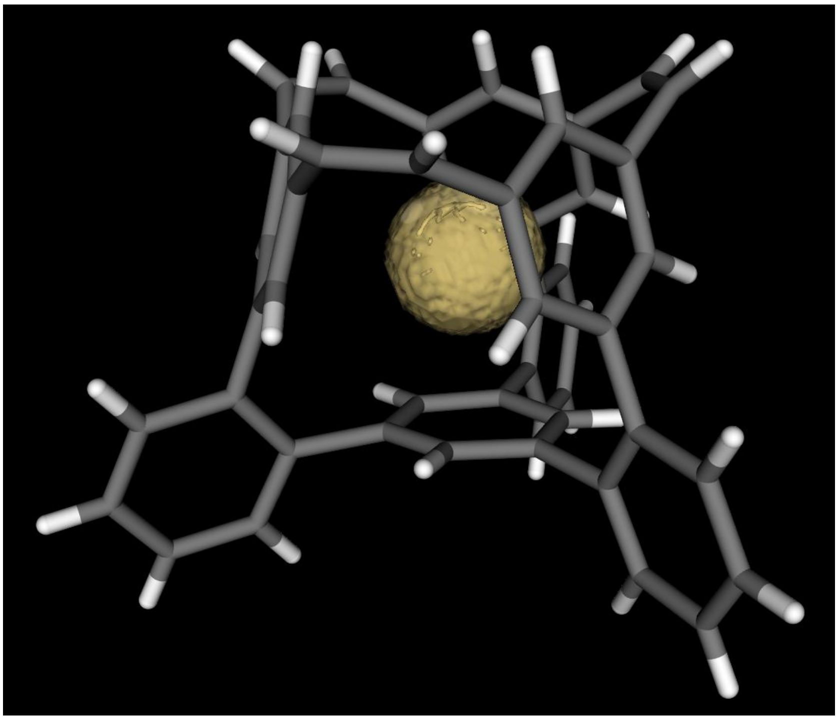

Figure S5. Probe occupied volume of the isolated cavity of **4** with a probe radius of 1.12Å.

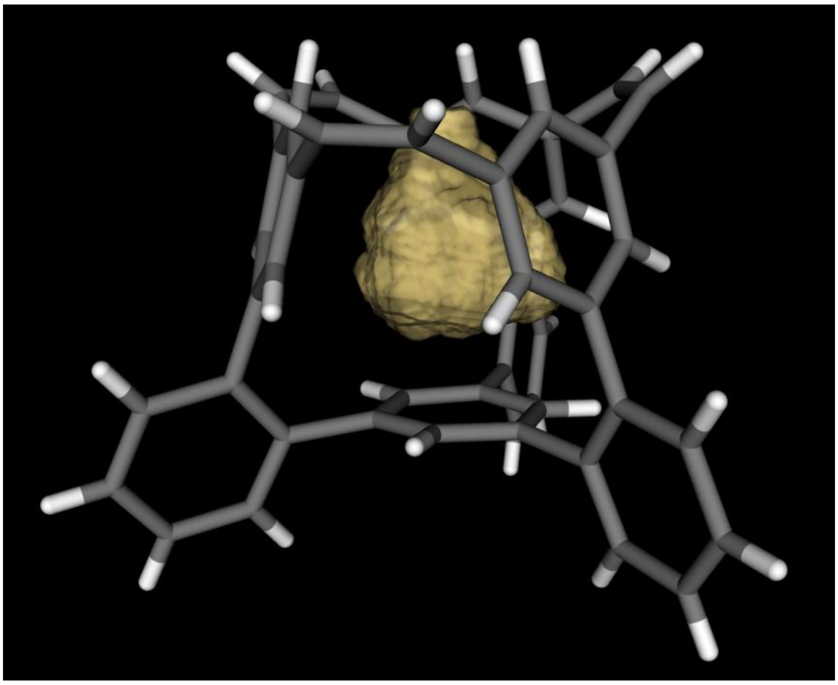

Figure S6. Probe occupied volume of the isolated cavity of **4** with a probe radius of 0.43Å.

5. **Quantum Chemical Calculations**

The Gaussian 16 program (Revision C.02)[4] running on the computing facilities "Taiwania 3" and "Forerunner 1" of the National Center for High-performance Computing, Taiwan, was used for all density functional theory (DFT) calculations. The molecular geometries were optimized using the B3LYP hybrid functional[5] in combination with the 6-31G(d) basis set. The XYZ files for the optimized structures are provided as supplementary material. Molecular orbitals are illustrated with an isovalue of 0.02. In addition, the Gauge-Independent Atomic Orbital (GIAO) method was used to calculate the nucleus-independent chemical shifts (NICS).[6] These calculations were performed in a single point fashion on geometries optimized at B3LYP/6-31G(d) level using the same functional and the larger 6-311+G(2d,p) basis set.

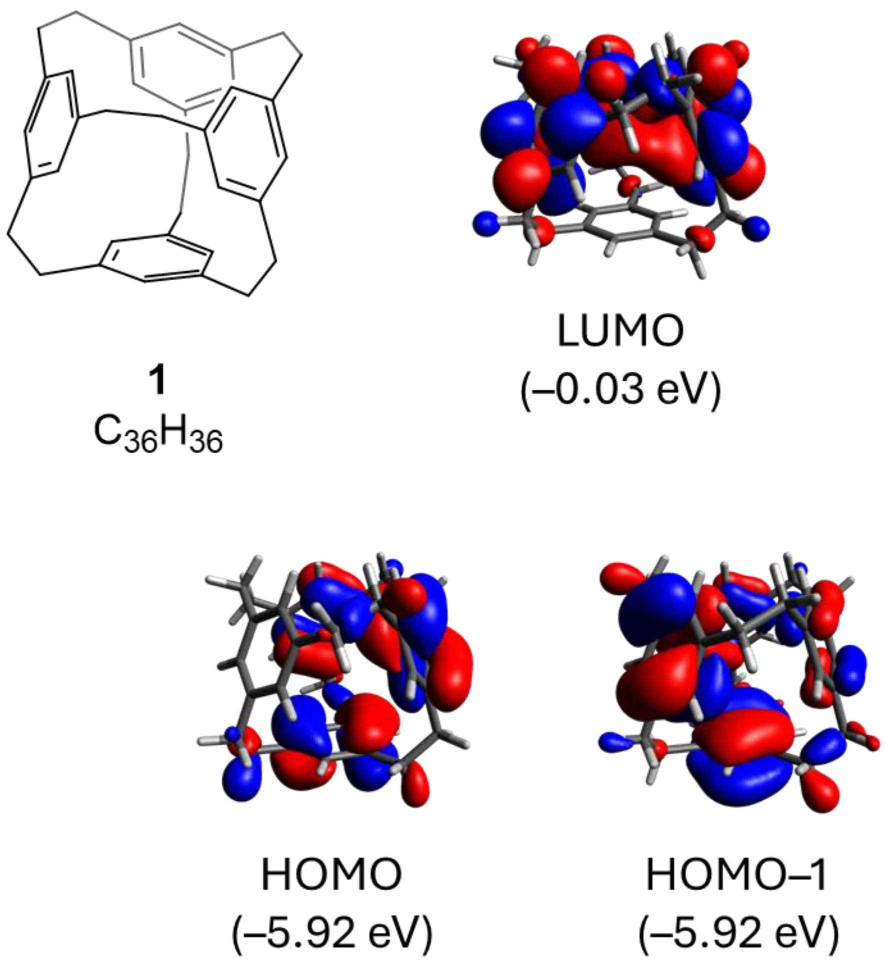

Figure S7. Frontier molecular orbitals of spheriphane **1**.

6. **Harmonic Oscillator Model of Aromaticity (HOMA) Analysis**

The HOMA values[7] were calculated based on the following equation:

$$\mathrm{HOMA} = 1 - \frac{\alpha}{n}\sum\left(R_{\mathrm{opt}} - R_{\mathrm{i}}\right)^2$$

Where α = 257.7 and R_{opt} = 1.388 were chosen as the reference parameters for CC bonds. R_{i} and n are the individual bond lengths and the total number of bonds taken into consideration, respectively.

The average values for each ring type were used for the calculations. Therefore, for ring A in **1** ($C_{36}H_{36}$), n = 24; for ring A in **2** ($C_{48}H_{36}$) and **4** ($C_{48}H_{30}$), n = 18; for ring B in **2** ($C_{48}H_{36}$) and **4** ($C_{48}H_{30}$), n = 18; for ring C in **2** ($C_{48}H_{36}$) and **4** ($C_{48}H_{30}$), n = 6.

All HOMA values for **1** ($C_{36}H_{36}$) and **2** ($C_{48}H_{36}$) were calculated based on B3LYP/6-31G(d) optimized geometries; while the HOMA values for **4** ($C_{48}H_{30}$) obtained from B3LYP/6-31G(d) optimized geometry and from its experimentally obtained crystal structure were presented separately.

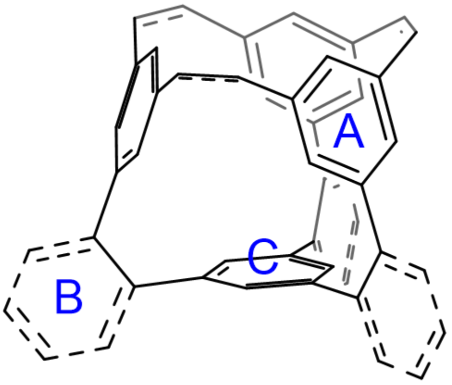

		HOMA		
		A	B	C
**1** ($C_{36}H_{36}$)		0.966	-	-
**2** ($C_{48}H_{36}$)		0.964	0.949	0.958
**4** ($C_{48}H_{30}$)	Calc.	0.959	0.951	0.956
	Expt.	0.989	0.983	0.987

Figure S8. HOMA values at different locations for **1**, **2**, and **4**.

7. **Reference**

(1) Mondal, B.; Ghosh, A. K.; Mukherjee, P. S. Reversible Multistimuli Switching of a Spiropyran-Functionalized Organic Cage in Solid and Solution. *J. Org. Chem.* **2017**, *82* (15), 7783–7790.

(2) Cui, S.; Zhuang, G.; Lu, D.; Huang, Q.; Jia, H.; Wang, Y.; Yang, S.; Du, P. A Three-Dimensional Capsule-like Carbon Nanocage as a Segment Model of Capped Zigzag [12,0] Carbon Nanotubes: Synthesis, Characterization, and Complexation with C_{70}. *Angew. Chem. Int. Ed.* **2018**, *57* (30), 9330–9335.

(3) Maglic, J. B.; Lavendomme, R. *MoloVol*: An Easy-to-Use Program for Analyzing Cavities, Volumes and Surface Areas of Chemical Structures. *J. Appl. Crystallogr.* **2022**, *55* (4), 1033–1044.

(4) Gaussian 16, Revision C.02, M. J. Frisch, G. W. Trucks, H. B. Schlegel, G. E. Scuseria, M. A. Robb, J. R. Cheeseman, G. Scalmani, V. Barone, G. A. Petersson, H. Nakatsuji, X. Li, M. Caricato, A. V. Marenich, J. Bloino, B. G. Janesko, R. Gomperts, B. Mennucci, H. P. Hratchian, J. V. Ortiz, A. F. Izmaylov, J. L. Sonnenberg, D. Williams-Young, F. Ding, F. Lipparini, F. Egidi, J. Goings, B. Peng, A. Petrone, T. Henderson, D. Ranasinghe, V. G. Zakrzewski, J. Gao, N. Rega, G. Zheng, W. Liang, M. Hada, M. Ehara, K. Toyota, R. Fukuda, J. Hasegawa, M. Ishida, T. Nakajima, Y. Honda, O. Kitao, H. Nakai, T. Vreven, K. Throssell, J. A. Montgomery, Jr., J. E. Peralta, F. Ogliaro, M. J. Bearpark, J. J. Heyd, E. N. Brothers, K. N. Kudin, V. N. Staroverov, T. A. Keith, R. Kobayashi, J. Normand, K. Raghavachari, A. P. Rendell, J. C. Burant, S. S. Iyengar, J. Tomasi, M. Cossi, J. M. Millam, M. Klene, C. Adamo, R. Cammi, J. W. Ochterski, R. L. Martin, K. Morokuma, O. Farkas, J. B. Foresman, and D. J. Fox, Gaussian, Inc., Wallingford CT, 2019.

(5) (a) Vosko, S. H.; Wilk, L.; Nusair, M. Accurate Spin-Dependent Electron Liquid Correlation Energies for Local Spin Density Calculations: A Critical Analysis. *Can. J. Phys.* **1980**, *58* (8), 1200–1211. (b) Lee, C.; Yang, W.; Parr, R. G. Development of the Colle-Salvetti Correlation-Energy Formula into a Functional of the Electron Density. *Phys. Rev. B* **1988**, *37* (2), 785–789. (c) Becke, A. D. Density-Functional Thermochemistry. III. The Role of Exact Exchange. *J. Chem. Phys.* **1993**, *98* (7), 5648–5652.

(6) (a) Schleyer, P. von R.; Maerker, C.; Dransfeld, A.; Jiao, H.; van Eikema Hommes, N. J. R. Nucleus-Independent Chemical Shifts: A Simple and Efficient Aromaticity Probe. *J. Am. Chem. Soc.* **1996**, *118* (26), 6317–6318. (b) Chen, Z.; Wannere, C. S.; Corminboeuf, C.; Puchta, R.; Schleyer, P. von R. Nucleus-Independent Chemical Shifts (NICS) as an Aromaticity Criterion. *Chem. Rev.* **2005**, *105* (10), 3842–3888.

(7) (a) Kruszewski, J.; Krygowski, T. M. Definition of Aromaticity Basing on the

Harmonic Oscillator Model. Tetrahedron Lett. 1972, 13 (36), 3839–3842. (b) Krygowski, T. M. Crystallographic Studies of Inter-and Intramolecular Interactions Reflected in Aromatic Character of. Pi.-Electron Systems. *J. Chem. Inf. Comput. Sci.* **1993**, *33* (1), 70–78. (c) Krygowski, T. M.; Cyrański, M. K. Structural Aspects of Aromaticity. *Chem. Rev.* **2001**, *101* (5), 1385–1420.

8. **NMR Spectra**

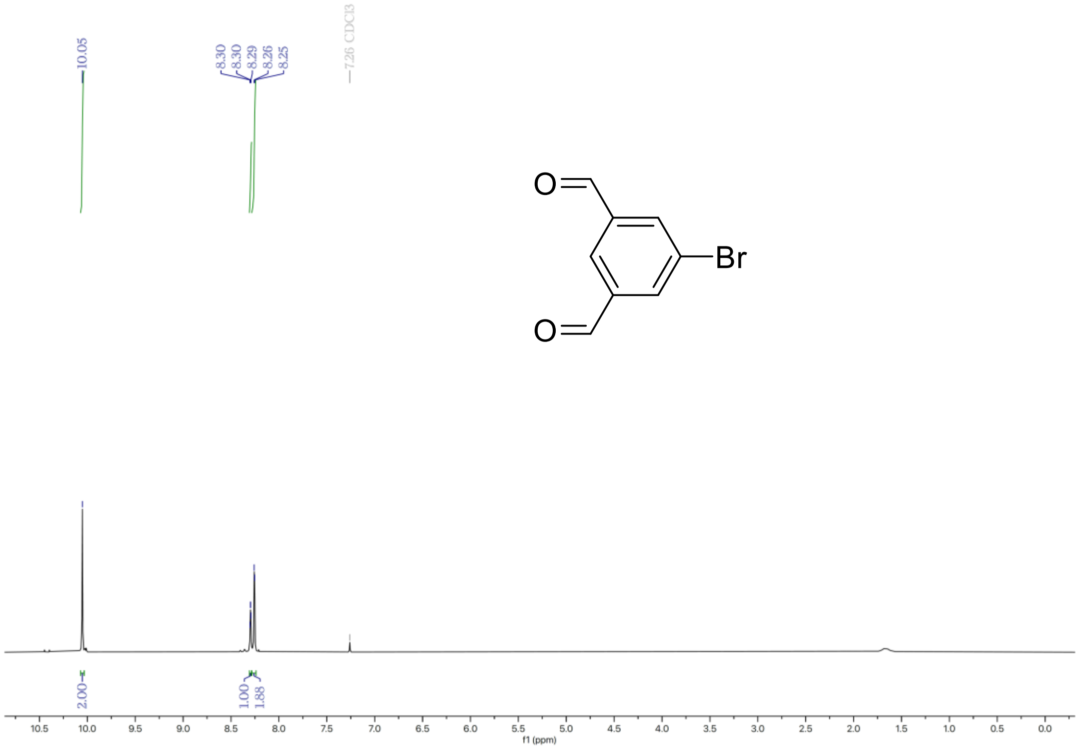

^{1}H NMR spectrum of **6** (300 MHz, $CDCl_3$)

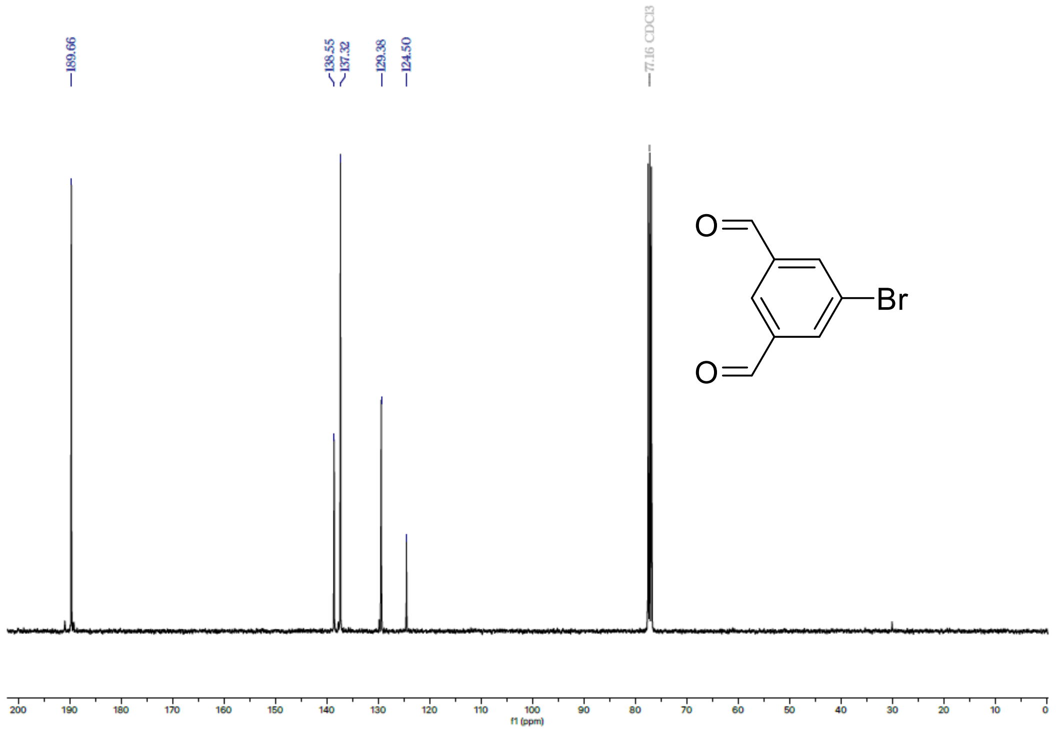

^{13}C NMR spectrum of **6** (100 MHz, $CDCl_3$)

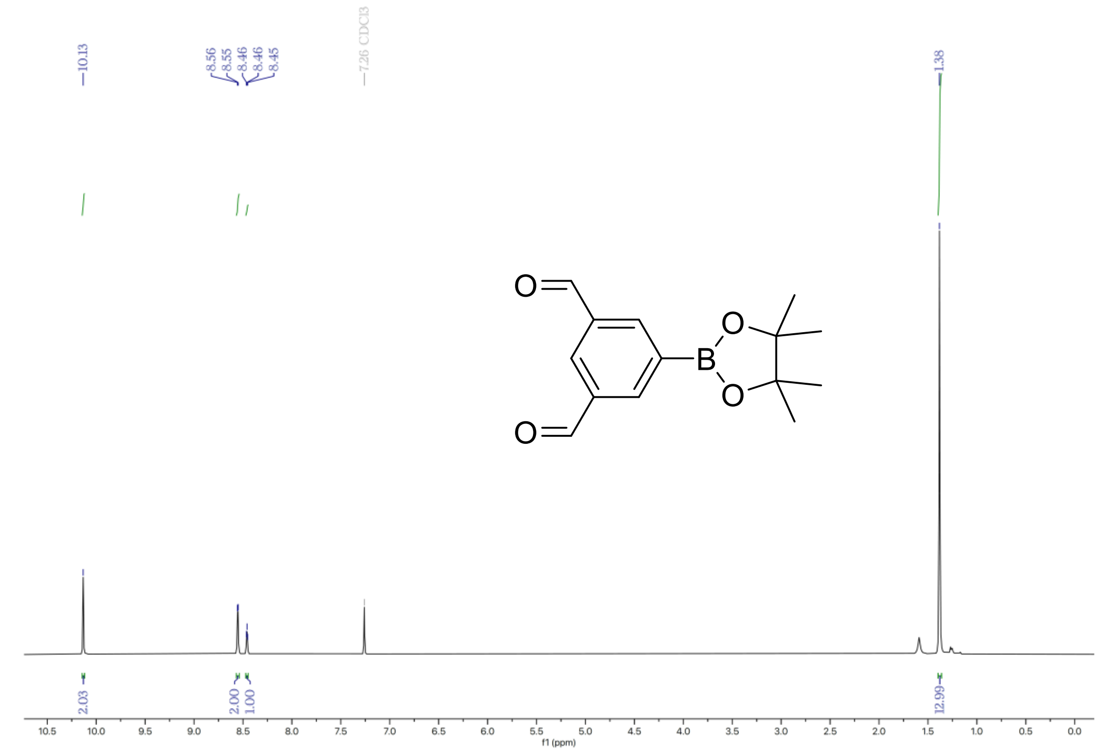

^{1}H NMR spectrum of **7** (300 MHz, $CDCl_3$)

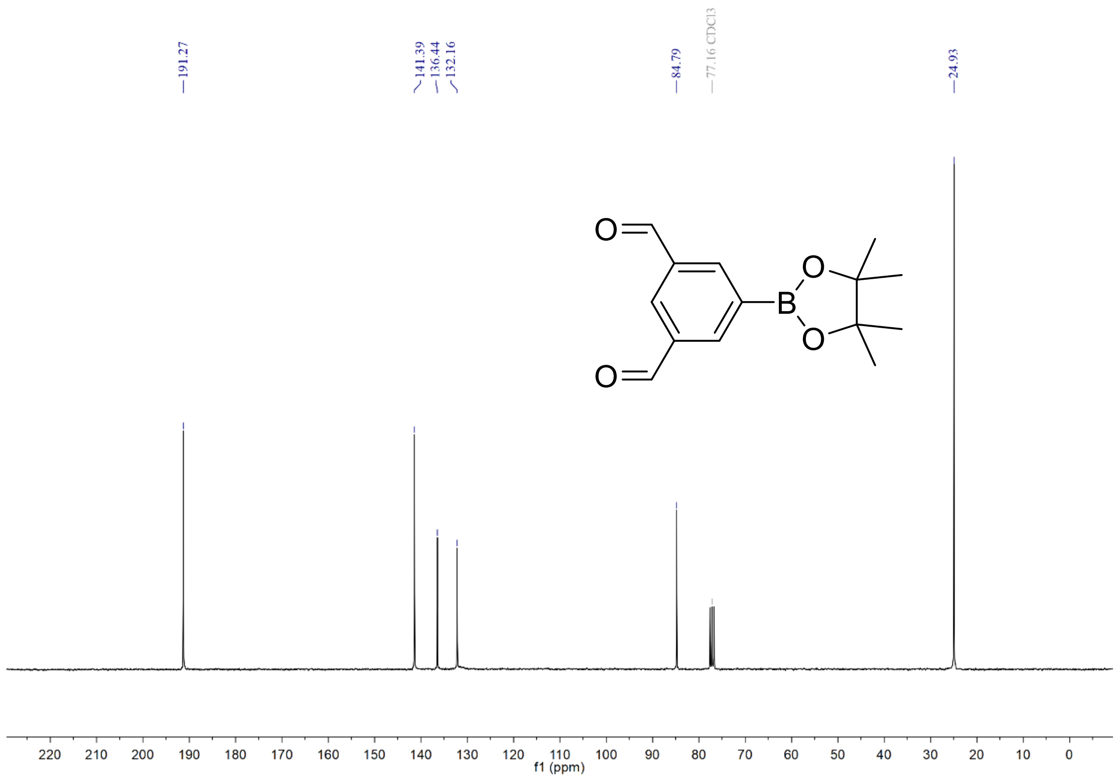

^{13}C NMR spectrum of **7** (100 MHz, $CDCl_3$)

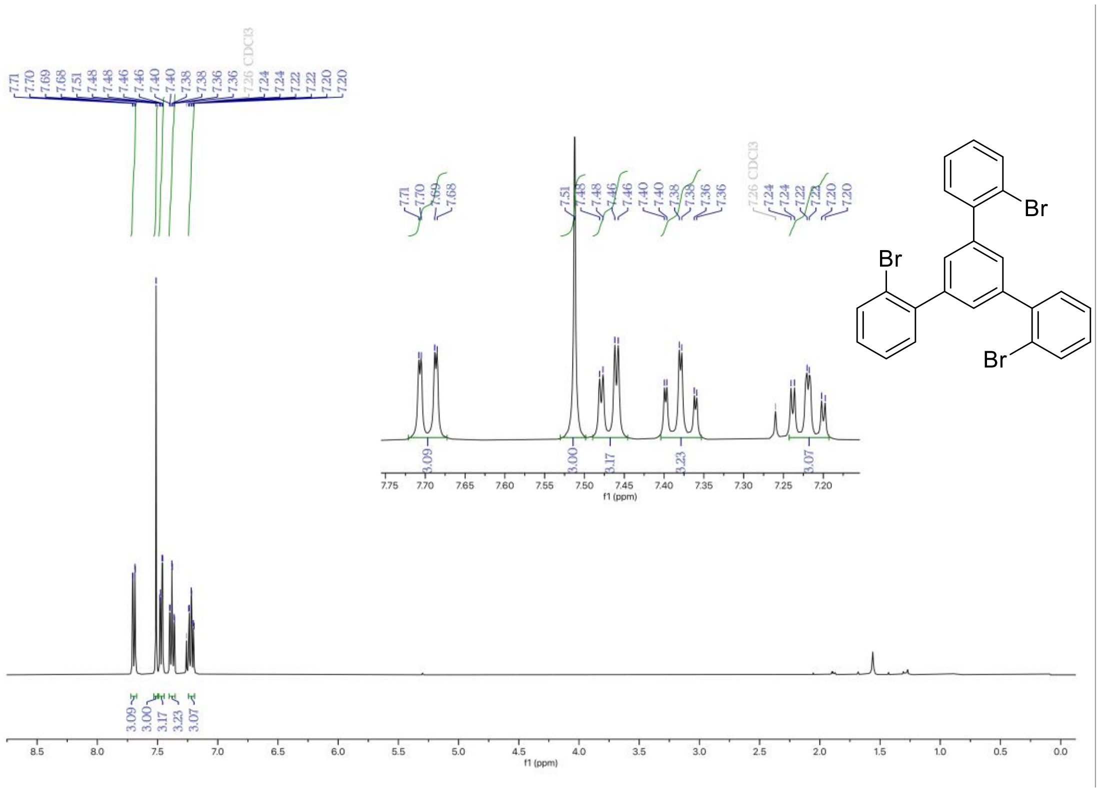

^{1}H NMR spectrum of **9** (400 MHz, $CDCl_3$)

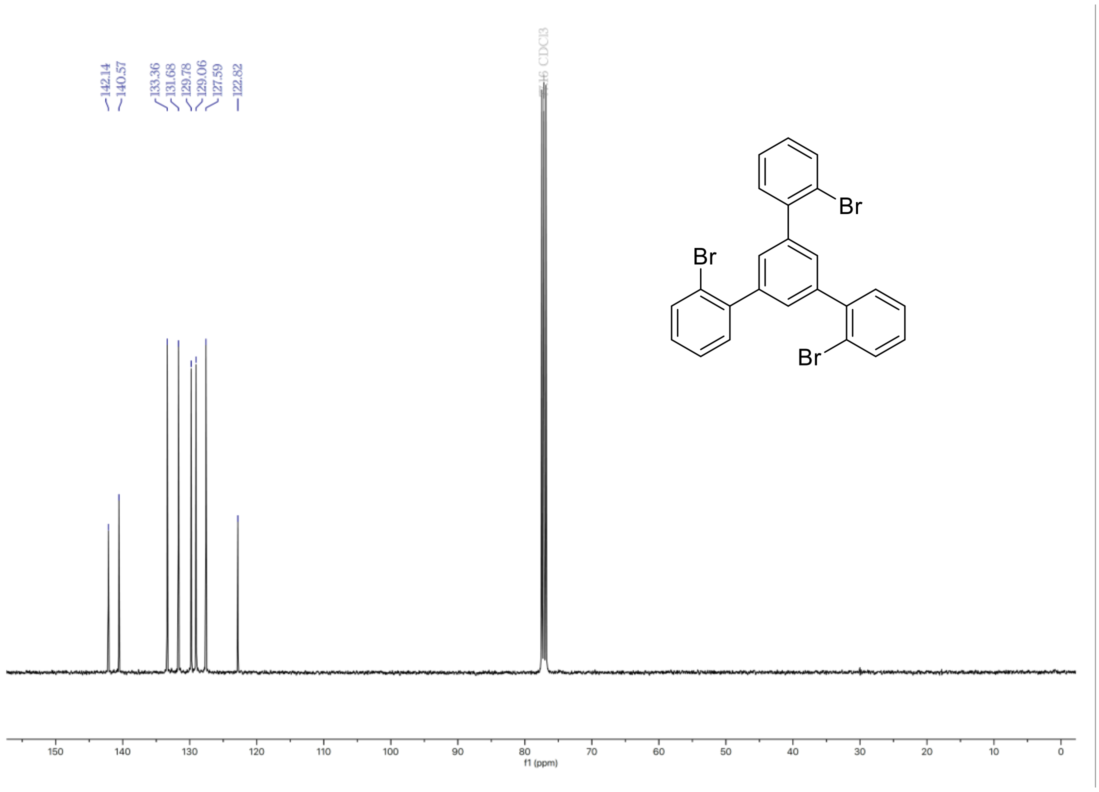

^{13}C NMR spectrum of **9** (100 MHz, $CDCl_3$)

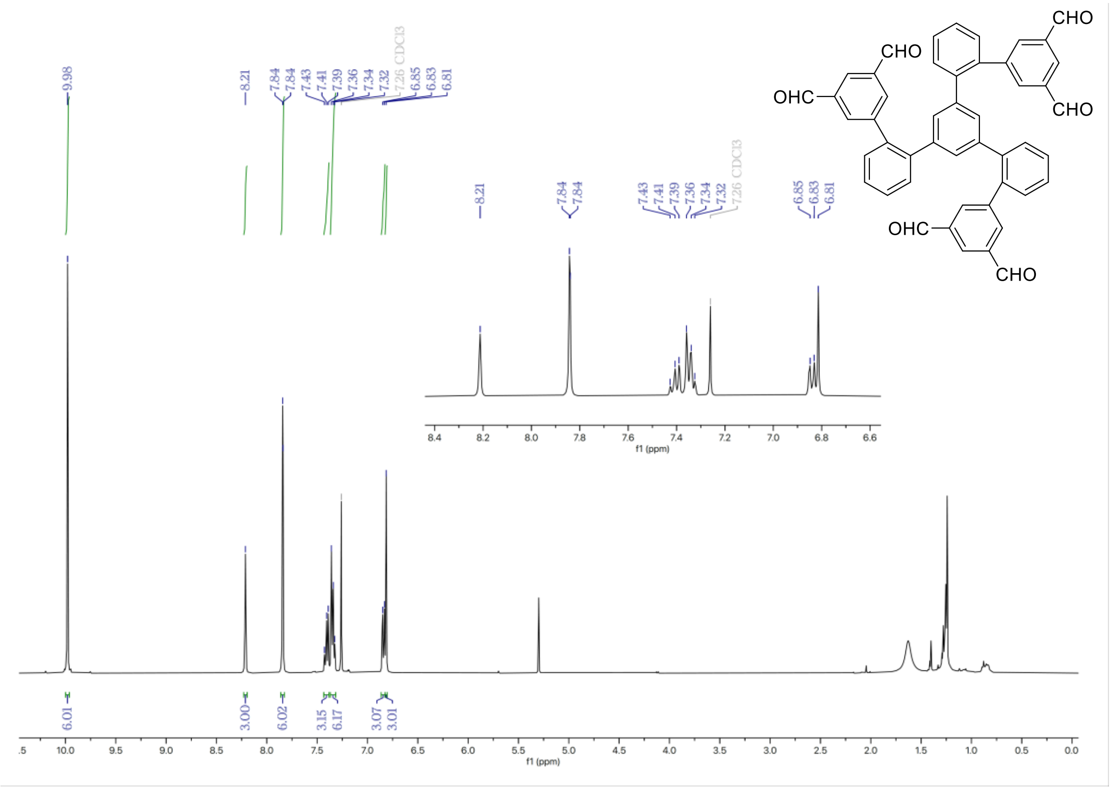

^{1}H NMR spectrum of **10** (400 MHz, $CDCl_3$)

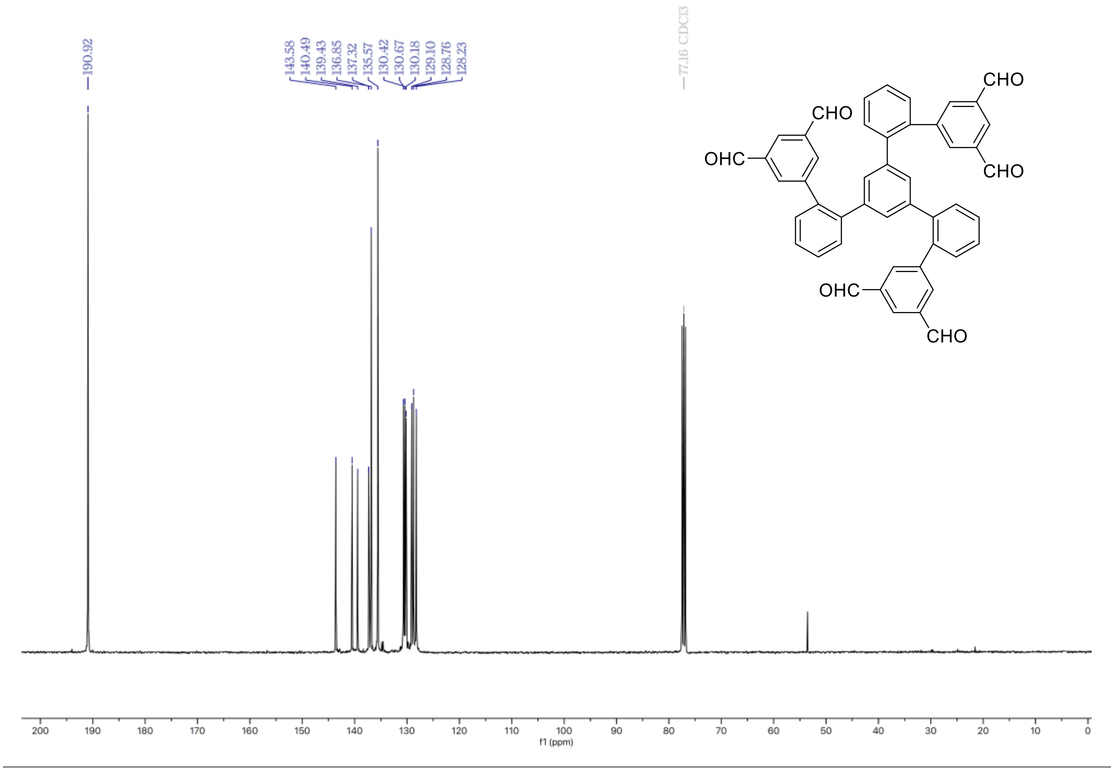

^{13}C NMR spectrum of **10** (100 MHz, $CDCl_3$)

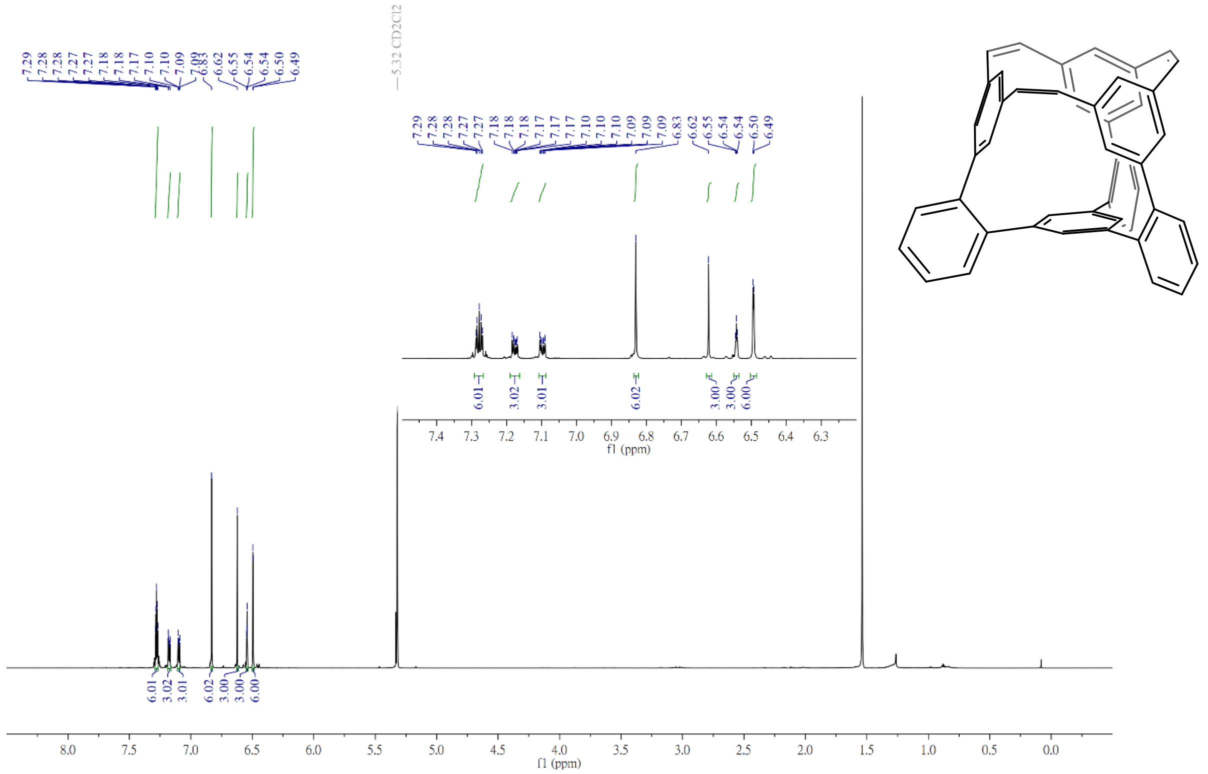

^1H NMR spectrum of **4** (600 MHz, CD_2Cl_2)

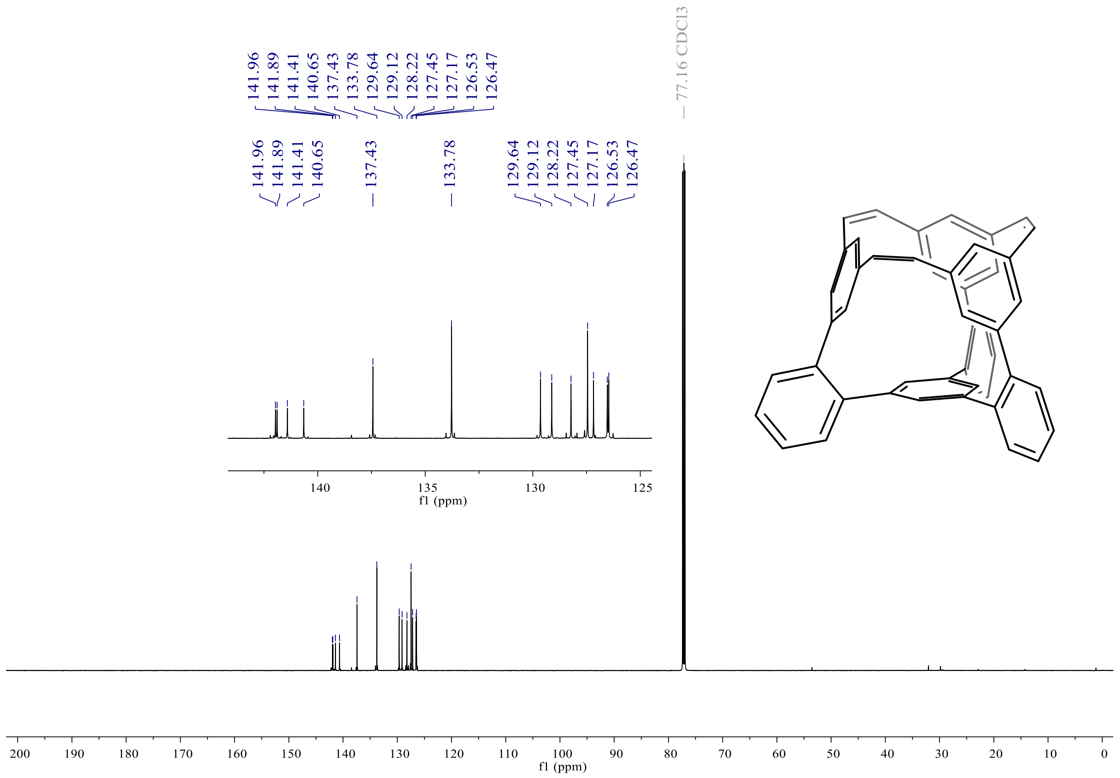

^{13}C NMR spectrum of **4** (150 MHz, $CDCl_3$)
